# Nurses’ perception of the hospitals’ culture and readiness of evidence-based practise integration in the hospital’s system in western Ethiopia

**DOI:** 10.1186/s12912-024-01741-2

**Published:** 2024-02-07

**Authors:** Dereje Chala Diriba, Temesgen Tilahun

**Affiliations:** 1 School of Nursing and Midwifery, Institute of Health Sciences, Wollega University, Nekemte, Ethiopia; 2 School of Medicine, Institute of Health Sciences, Wollega University, Nekemte, Ethiopia

**Keywords:** Evidence-based practise, Culture and readiness, Nurses, Ethiopia

## Abstract

**Background:**

Evidence-based practise is a method by which healthcare professionals integrate the best available evidence, individual expertise and patient preferences to improve patient safety and utilise quality healthcare. No study was conducted in Ethiopia to assess the nurses’ perception of the hospitals’ culture and readiness for evidence-based practice integration into the hospital’s system. Hence, this study aimed to assess the perception of nurses on the hospitals’ culture and readiness of evidence-based practise integration in hospitals’ systems in Western Ethiopia.

**Methods:**

A cross-sectional study involving 412 nurses in six hospitals in western Ethiopia was conducted between December 2022 and February 2023. A 25-item Organisational Culture and Readiness of System-Wide Integration of EBP scale was used, with a Cronbach’s alpha of 0.94. While 25 is the minimum score, 125 is the maximum; higher scores indicate better hospital culture and readiness for system-wide integration of evidence-based practice. A self-administered data collection technique was used. Descriptive statistics were computed using Statistical Package for the Social Sciences version 25 software.

**Results:**

Four hundred and twelve nurses participated in the study. The majority (85.9%) were bachelor’s degree holders and over a third (34.7%) worked in primary hospitals. Only a quarter (26.5%) had ever received mentorship from their leader on implementing evidence-based practice. The overall hospital culture and readiness score for system-wide integration of evidence-based practice among nurses in six hospitals was 70.3 ± 17.3.

**Conclusion:**

The nurses’ perception of the hospitals’ culture and readiness score for system-wide integration of evidence-based practice in six hospitals in Western Ethiopia was equivocal. There is a need to engage all resources and increase leadership commitment to make evidence-based practice a hospital culture. Further research is warranted to understand the national hospitals’ status in establishing and sustaining evidence-based practise culture.

## Introduction

Evidence-based practice (EBP) is a practical decision-making process to promote health and improve patient safety [1]. The National Institute of Corrections defined EBP as “the objective, balanced, and responsible use of current research and the best available data to guide policy and practise decisions, such that outcomes for consumers are improved” [2]. In addition to its importance in clinical practice to improve patient safety, there are varieties in its application and integration of EBP in health care. Even though different countries, including Ethiopia, have implemented EBP, the institutionalisation of EBP in the health system is lacking [3]. The Ministry of Health of Ethiopia initiated continuous professional development for health professionals working in a clinical setting to update their knowledge of different guidelines and current evidence. However, there is a gap in the knowledge, attitudes and implementation of EBP. Recent studies have reported insufficient knowledge and implementation of EBP [4, 5], suggesting the need to improve the knowledge and implementation of EBP.

All healthcare professionals should implement EBP; however, nurses play a vital role in implementing EBP to boost patient care outcomes and safety. Clinicians have a distinctive occasion to enact the system-wide integration of EBP [6]. Factors related to nurses and organisational readiness relate to facilitating EBP in health care systems. Nurses’ attitudes and beliefs are correlated with the implementation of EBP [7]. Some nurses, including their leaders, claim time constraints, lack of research experience, and lack of access to research databases, and half of the nurses do not know how to measure the outcomes [5]. A study showed that the organisational culture and readiness for EBP were low, and low priority was given [5]. To facilitate EBP, job performance evaluation, integration of nurses in decision-making, educational support to implement EBP, and educational outreach visits are necessary [8]. Making EBP culture and integrating these factors in health care systems is needed. A qualitative study involving leaders from four countries demonstrates that national contextual policies and organisational and service delivery influences are required to implement EBP. In addition, the challenges of EBP should be identified and resolved [9].

Organisational culture and situation can promote or hinder EBP in a system-wide integration, which claims different facilitators and barriers. The barriers to implementing EBP include inadequate resources and infrastructures, lack of leadership support, insufficient EBP knowledge and skills, lack of education and skills-building opportunities, and EBP mentors [10]. Apart from these barriers, the culture of EBP directly influences EBP knowledge, beliefs, competency, mentoring, and job satisfaction. EBP mentorship also enhances EBP competency and implementation and nurses’ intent to stay in their positions [10]. Hence, organisational culture and readiness are needed to implement EBP in healthcare settings. However, the implementation of EBP is still not standard in healthcare systems worldwide, including in Ethiopia [10, 11]. Nearly half of the nurses incorporated evidence in their clinical practices [11, 12]. A mixed-method study conducted in Northwest Ethiopia showed that education level, administrative support, attitude toward EBP and availability of information sources influence the implementation of EBP [11, 13]. No study was conducted in Ethiopia to assess the nurses’ perception of the hospitals’ culture and readiness for EBP integration into the hospital’s system. Hence, this study aimed to assess nurses’ perceptions of hospitals’ culture and readiness for EBP integration in hospital systems in Western Ethiopia.

## Methods

### Study design

An institution-based descriptive cross-sectional study was conducted.

### Study setting and period

The study was conducted in six public hospitals located in Western Ethiopia, including Wollega University Referral Hospital (teaching hospital) (121 nurses), Nekemte Specialised Hospital (130 nurses), Sire Primary Hospital (54 nurses), Arjo Primary Hospital (52), Bako Primary Hospital (52 nurses) and Gimbi General Hospital (66 nurses). A total of 475 nurses were working in these hospitals. These public hospitals serve people from four zones in Western Ethiopia. Subjects were recruited in the hospitals, and the data were collected between December 2022 and February 2023.

### Participants

The participants were nurses working in the included hospitals located in western Ethiopia. Nurses were included in the study if they (a) had completed at least a diploma in nursing and (b) had more than six months of work experience in that hospital. The participants were excluded if they did not volunteer to participate in the study and were on maternity leave.

### Variables

The study variables include the level of nurses’ perception of the culture and readiness of the hospital to integrate EBP; sociodemographic characteristics, including age, gender, ethnicity, religion, education level, roles, organisation, and the year since completing the last study; and professional characteristics, such as accessibility to the internet, training since last year, mentorship on EBP and research-related training.

### Data measurements

The perception of nurses on the hospitals’ culture and readiness for EBP integration in hospitals’ systems was assessed using an Organisational Culture and Readiness Scale for System-Wide Integration of Evidence-Based Practise (OCRSIEP) scale. The scale had excellent internal reliability (above 0.85) and had established face, content and factorial validities [14, 15]. The Cronbach alpha of the scale used in this study is 0.94. There are 25 items on the scale, and each item is scored using a 5-point Likert scale (1 = none at all, 2 = a little, 3 = somewhat, 4 = moderately, and 5 = very much). Item totals are summed, with scores ranging from 25 to 125 [15]. Higher scores indicate stronger cultures and readiness for EBP within an organisation. The tool was adapted without translation because the respondents were nurses and were believed to understand the questionnaire. The authors developed questions regarding sociodemographic and professional characteristics.

### Data collection methods

Data were collected using a self-administered survey using a hard copy. Eight nonnurse-trained data collectors facilitated the data collection. On average, data collection took two weeks in each hospital.

### Sample size determination

**Fig. 1:**
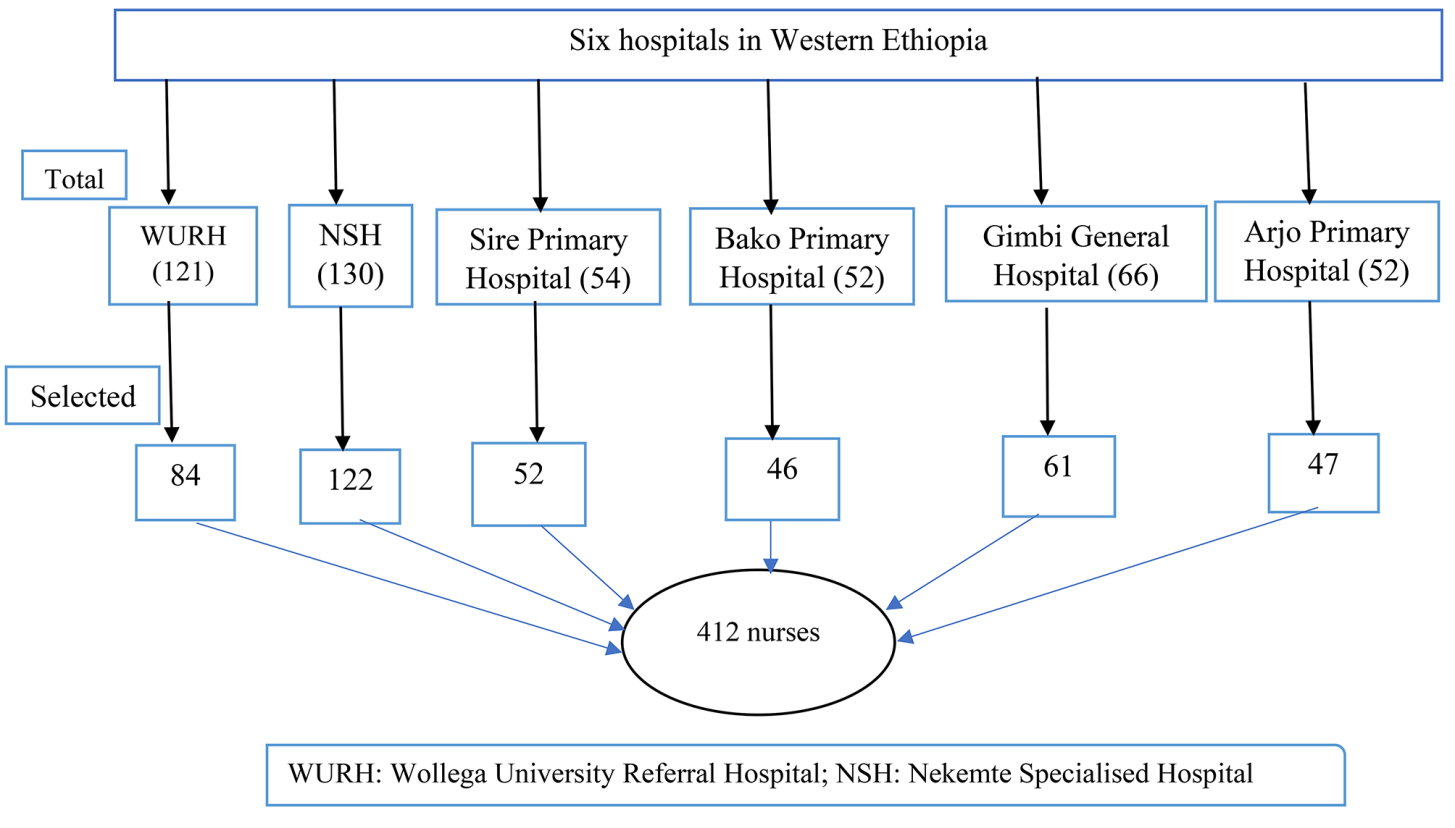
The sampling technique used to select the study participants

All eligible nurses working in the selected hospitals were included in the study to ensure the adequacy of the samples. Nurses working in Wollega University Referral Hospital (84), Nekemte Specialised Hospital (122), Sire Primary Hospital (52), Bako Primary Hospital (46), Arjo Primary Hospital (47) and Gimbi General Hospital (61) were included in the study. Among eligible samples, 32 were on maternity leave, and 31 refused to participate in the study (Fig. 1).

### Sampling techniques

The list of the nurses was obtained from the nurses’ metron office. All eligible nurses were recruited. Figure 1 depicts the sampling techniques used to select eligible participants.

### Statistical analysis

Data were entered and analysed using SPSS version 25 software. The missing data pattern was analysed before analyses, and multiple imputations were made. Descriptive statistics summarised the participants’ sociodemographic and professional characteristics and the perception of nurses on the hospitals’ culture and readiness of EBP integration in the hospitals’ system.

## Results

### Sociodemographic characteristics of the participants

Table 1 reveals the sociodemographic characteristics of the participants. Four hundred twelve nurses participated in the study, showing a 92.8% (412/444) response rate. The mean age of the participants was 30.6 ± 6.2 years. More than half (54.4%) were males. The majority (85.9%) were bachelor’s degree holders, and the mean number of years since participants completed their last education program was 6.2 ± 3.9 years. Over one-third (34.7%) worked in primary hospitals, and over three-fourths (80.6%) were staff nurses.

**Table 1:** Sociodemographic characteristics of the study participants in hospitals in western Ethiopia (*n* = 412)

Variables	Categories	Frequency (%)/Mean ± SD
Age	Age (in years)	30.6 ± 6.2
Gender	Male Female	224 (54.4%) 188 (45.6%)
Ethnicity	Oromoo Amhara	406 (98.5%) 6 (1.5%)
Religion	Christian (Protestant) Christian (Orthodox)	324 (78.6%) 88 (21.4%)
Education level	Diploma Bachelor Masters	44 (10.7%) 354 (85.9%) 14 (3.4%)
The year since the last education program completion	Duration (in years)	6.2 (3.9)
Hospital level	Primary General Specialised Teaching	143 (34.7%) 61 (14.8%) 124 (30.1%) 84 (20.4%)
Role of nurses	Staff nurses Head nurses Metron/coordinator/deputy metron	332 (80.6%) 65 (15.8%) 15 (3.6%)

Table 2 indicates the professional characteristics of the nurses. A third (33.0%) of nurses had received any updated training in the last year. Less than one-fifth (16.3%) had ever taken research-related training, and more than half (55.8%) had internet access in the hospital. Only a quarter (26.5%) had ever received any mentorship from their leader on the implementation of EBP.

**Table 2:** Professional characteristics of the participants in hospitals in western Ethiopia (*n* = 412)

Variables	Categories	Frequency (%)/Mean ± SD
Have you ever taken any updated training in the last year?	Yes No	136 (33.0%) 276 (67.0%)
Have you ever taken any research-related training?	Yes No	67 (16.3%) 345 (83.7%)
Do you have access to the internet in the hospital?	Yes No	230 (55.8%) 182 (44.2%)
Have you ever received any mentorship from your leader on implementing EBP?	Yes No	109 (26.5%) 303 (73.5%)

### Nurses’ perception of the hospitals’ culture and readiness of system-wide integration of EBP

Table 3 indicates the nurses’ perception level of the hospitals’ culture and readiness for system-wide integration of EBP. The mean nurses’ perception of the hospitals’ Culture and Readiness score for System-Wide Integration of EBP in six hospitals was 70.3 ± 17.3. The higher scores of hospitals’ culture and readiness to integrate EBP in their system were attributed to nurses’ beliefs that EBP is practised in their hospital (80.2) and the commitment of nurses (84.3) and physicians (81.1) to practise EBP. Nurses working in teaching hospitals better perceived the hospitals’ culture and readiness scores for the system-wide integration of EBP.

The lower score in hospitals’ culture and readiness to integrate EBP was attributed to a lack of nurse scientists (doctorally prepared researchers) in the organisation to assist in the generation of evidence when it does not exist, limited access to quality computers and access to electronic databases for searching for the best evidence, lack of library or limited librarian knowledge and skills of EBP. Moreover, limited librarians’ attention to the search for evidence and inadequate fiscal resources used to support EBP were also stated as factors influencing the hospital’s culture and readiness to integrate EBP.

**Table 3:** Nurses’ perception level of the hospitals’ culture and readiness for system-wide integration of EBP in hospitals in western Ethiopia (*n* = 412)

S/N	Item	Mean (SD) score
	The overall level of OCRSIEP	70.3 (17.3)
1.	To what extent is EBP clearly described as central to the mission and philosophy of your institution?	76.4 (26.5)
2.	To what extent do you believe that EBP is practiced in your organisation?	80.2 (26.2)
3.	To what extent is the nursing staff with whom you work committed to EBP?	84.3 (25.7)
4.	To what extent is the physician team with whom you work committed to EBP?	81.1 (25.9)
5.	To what extent are there administrators within your organisation committed to EBP (i.e., have planned for resources and support [e.g., time] to initiate EBP)?	74.5 (25.8)
6.	In your organisation, to what extent is there a critical mass of nurses who have strong EBP knowledge and skills?	77.3 (26.6)
7.	To what extent are there nurse scientists (doctorally prepared researchers) in your organisation to assist in generation of evidence when it does not exist?	58.2 (30.0)
8.	In your organisation, to what extent are there Advanced Practiced Nurses who are EBP mentors for staff nurses as well as other APNs?	66.3 (28.8)
9.	To what extent do practitioners model EBP in their clinical settings?	69.1 (26.5)
10.	To what extent do staff nurses have access to quality computers and access to electronic databases for searching for best evidence?	58.8 (27.6)
11.	To what extent do staff nurses have proficient computer skills?	61.6 (23.8)
12.	To what extent do librarians within your organisation have EBP knowledge and skills?	59.4 (27.0)
13.	To what extent are librarians used to search for evidence?	57.6 (26.6)
14.	To what extent are fiscal resources used to support EBP (e.g. education-attending EBP conferences/workshops, computers, paid time for the EBP process, mentors)	57.2 (27.5)
15.	To what extent are there EBP champions (i.e., those who will go the extra mile to advance EBP) in the environment among:	
	a. Administrators? b. Physicians? c. Nurse Educators? d. Advance Nurse Practitioners? e. Staff Nurses?	66.3 (28.3) 67.4 (27.1) 67.4 (29.0) 67.8 (28.4) 70.0 (28.1)
16.	To what extent is the measurement and sharing of outcomes part of the culture of the organisation in which you work?	71.0 (25.1)
17.	To what extent are decisions generated from:	
	a. direct care providers? b. upper administration? c. physician or other healthcare provider groups?	74.6 (26.6) 79.6 (26.4) 79.7 (25.6)
18.	Overall, how would you rate your institution in readiness for EBP	75.7 (25.9)
19.	Compared to 6 months ago, how much movement in your organisation has there been toward an EBP culture. (place a hatch mark on the line to the right that indicates your response)	75.3 (26.9)
	OCRSIEP by hospital level	
	Primary	69.8 (15.3)
	General	65.9 (15.6)
	Specialised	71.9 (17.1)
	Teaching	73.5 (21.1)

EBP: Evidence-based practise, OCRSIEP: Organisational Culture and Readiness Scale for System-Wide Integration of Evidence-Based Practise

## Discussion

This study assessed the nurses’ perception of the hospitals’ culture and readiness of system-wide integration of EBP in western Ethiopia. The hospital culture and readiness to integrate EBP into their system are mandatory to improve healthcare. A key finding was that nurses perceived slightly more than half of the overall score regarding a hospital culture and readiness to integrate EBP into the hospital’s system. According to nurses’ perception, there is a better movement towards a sustainable culture and readiness to implement EBP in the teaching hospital.

Nurses perceived the hospitals’ culture and readiness to integrate EBP into the hospital’s system as 70.3. This finding is comparable with the finding from Ireland, which reported that clinicians’ perceptions of organisational support and readiness for EBP was 74.07 [6]. The result is low compared with a study conducted in the United States involving educators and clinical staff of teaching hospitals [16]. This might be due to the increased utilisation of evidence in teaching institutions by students and educators [6]. Different factors can be considered in enhancing readiness to establish and sustain a culture of EBP in the hospital, such as a lack of doctorally prepared nurses. In Ethiopia, no nurse scientists prepared at the doctorate level at the hospitals that mentored the nurses on EBP implementation. The limited number of doctorate-level nurses are only available in the university as educators. This finding collaborates with a study in Ireland, which reported insufficient EBP champions or mentors as a challenge to engage EBP in the hospitals [6]. Another study in the United States found the organisation’s culture, the structure of the nurse’s leadership, and resources in the hospital as facilitators or barriers to empowering nurses under their mentorship to use EBP [8]. Thus, it is suggested to increase the educational career of nurses up to the doctoral level to immerse EBP as a culture of hospitals [17]. Nurses perceive that the availability of institutional resources, prioritisation of resources and expectations of EBP implementation support the implementation of EBP [8]. In this study, the institutional resources that help the implementation of EBP are still lacking/inadequate, including a lack of computers, financial support for EBP conferences and paid time for the EBP process.

Furthermore, the leaders did not arrange updates and research methods training. A pilot experimental study reported that a conducive environment and the availability of necessary resources to support evidence-based practice are needed [18]. Hence, hospital managers are suggested to establish and sustain internet access and other resources to boost EBP. Mentorship culture is reported to motivate nurses to implement EBP [8]. However, the number of nurses who received mentorship in their practice was low. Hence, a mentorship culture is needed to enhance and motivate nurses to implement EBP.

Beyond institutional resources, the nurses reported a limited library, lack of databases, limited EBP knowledge and skills of librarians and low intention to search for evidence. A qualitative study in the United States mentioned that nurses need library resources such as computers, databases, evidence, and the internet [8]. Another study conducted in Ireland reported that the underutilisation of librarians was raised as a challenge in the implementation of EBP [6]. Hence, library resources should be available to improve the hospital’s EBP culture. Librarians should be updated on EBP knowledge and skills and increase their utilisation to help nurses search for evidence. The strength of this study was the involvement of large samples. A self-administered data collection method was used, which could cause response bias. Another limitation of the study was that the scale used was not tested for its validity in Ethiopia.

## Conclusion

Even though some hospitals included evidence-based practice in their mission and philosophy, integrating evidence-based practice in the hospital system in western Ethiopia was equivocal. Nurses and physicians were committed to integrating evidence-based practice into the hospital’s system. Hospital resources, continuous professional updates, research methods training, and improving librarians’ evidence-based practise knowledge and skills are mandatory to integrate better evidence-based practise into the hospital system. Future studies should involve more nurses from all corners of the country, and the views of other health professionals are needed.

## Data Availability

The datasets analysed during this study are available from the corresponding author upon reasonable request.

## Abbreviations

APN: Advanced practised nurse
EBP: Evidence-based practice
OCRSIEP: Organisational Culture and Readiness Scale for System-Wide Integration of Evidence-Based Practise
SD: Standard deviation
